# Extended Phylogeny and Extraintestinal Virulence Potential of Commensal *Escherichia coli* from Piglets and Sows

**DOI:** 10.3390/ijerph17010366

**Published:** 2020-01-06

**Authors:** Ewa Bok, Aleksandra Kożańska, Justyna Mazurek-Popczyk, Magdalena Wojciech, Katarzyna Baldy-Chudzik

**Affiliations:** 1 Department of Microbiology and Molecular Biology, Collegium Medicum, University of Zielona Góra, 65-561 Zielona Góra, Poland; j.mazurek-popczyk@cm.uz.zgora.pl (J.M.-P.); k.baldy-chudzik@cm.uz.zgora.pl (K.B.-C.); 2 Department of Biotechnology, Faculty of Biological Sciences, University of Zielona Góra, 65-561 Zielona Góra, Poland; ola19937@onet.eu; 3 Department of Mathematical Statistics and Econometrics, Faculty of Mathematics, Computer Science and Econometrics, University of Zielona Góra, 65-516 Zielona Góra, Poland; m.wojciech@wmie.uz.zgora.pl

**Keywords:** commensal *Escherichia coli*, piglets, sows, virulence genes (VGs), phylogenetic typing, nonlinear mixed models

## Abstract

Commensal *Escherichia coli*, naturally occurring in the intestinal tract, can be the origin of extraintestinal pathogenic *E. coli* (ExPEC) strains. ExPEC causes high mortality and significant economic losses in the swine industry in several countries and poses a serious threat to public health worldwide. The aim of this study was to analyze the extended phylogenetic structure and extraintestinal virulence potential in two groups of commensal *E. coli* isolates from post-weaning piglets and sows. The phylogenetic assignment to eight groups was determined using the revised Clermont phylogenetic typing method in quadruplex PCR. Identification of extraintestinal virulence genes (VGs) and adhesin operon genes was performed using multiplex or simplex PCR. The revised phylogenetic assignment allowed us to distinguish *E. coli* with significantly higher (groups C and F) or lower (group E) virulence potential in isolates from piglets. The majority of the tested VGs occurred more frequently in isolates from piglets than from sows, with statistically significant differences for seven genes: *fimH*, *papAH*, *iutA*, *iroN*, *ompT*, *traT*, and *iss*. Complete operons for type I and P fimbriae significantly prevailed among *E. coli* from piglets. This study provides insight into the extended phylogenetic structure of porcine commensal *E. coli* and showed that these strains, particularly from piglets, constitute a considerable reservoir of extraintestinal VGs and may increase the potential risk of extraintestinal infections.

## 1. Introduction

Commensal *Escherichia coli* strains are members of the facultative anaerobic natural microbiota of humans and animals [1]. These bacteria are usually harmless, but part of their population can become extraintestinal pathogenic *E. coli* (ExPEC). ExPEC have a fecal origin, occur asymptomatically in the intestinal tract, and sometimes can colonize extraintestinal niches and cause serious diseases [2,3]. Typical extraintestinal infections include urinary tract infections (UTI), pyelonephritis, sepsis, pneumonia, and meningitis [4,5,6]. The virulence potential of ExPEC depends on the various extraintestinal virulence-associated factors in bacteria–host interactions, rather than a simple mechanism [7]. Characteristic ExPEC virulence factors include various adhesins (type I and P fimbriae), iron acquisition and utilization systems (aerobactin and salmochelin siderophores), protectins (structural components of the bacterial outer membrane), toxins (hemolysin, cytotoxic necrosis factor), and biofilm formation factor (antigen 43). These virulence factors facilitate colonization and invasion of the host, as well as avoidance or disruption of host defense mechanisms [8,9]. ExPEC strains were defined by Johnson et al. [10] as *E. coli* isolates containing two or more of the following virulence markers: *papA/papC*, *sfa/foc*, *afa/dra*, *kpsMTII*, and *iutA*. Virulence genes (VGs) responsible for pathogenicity are usually encoded on pathogenicity islands (PAIs), plasmids, and other mobile genetic elements, and can thus be transmitted via horizontal gene transfer (HGT) between various *E. coli* strains [2]. Commensal or pathogenic bacteria may, in a single HGT event, acquire a mobile genetic element carrying multiple VGs, antimicrobial-resistance genes, or other genes encoding features that offer a niche advantage [11,12]. Many studies have suggested a strong association between virulence gene carriage and the phylogenetic type of *E. coli* strains [7,13,14].

The population structure of *E. coli* is predominantly clonal and strains can be classified into one of eight phylogenetic groups: A, B1, B2, C, D, E, F, and cryptic clade I [15]. In humans, commensal *E. coli*, with no pathogenic features, most often represent group A or B1, whereas the majority of *E. coli* responsible for extraintestinal infections are classified into B2 and D phylogroups. Group E is related to group D, while group F is related to the main group, B2 [1,7]. Factors that shape the phylogenetic structure of *E. coli* are: environment, gut anatomy, physiology, and diet. In animals, the main environmental force influencing the phylogenetic structure of the *E. coli* is the domestication status of the host [16]. Domesticated animals have a lower proportion of B2 strains than their wild counterparts and a higher proportion of A strains [17]. In the phylogenetic structure of the commensal *E. coli* strains derived from pigs, phylogroup A dominates, followed by group B1. Strains classified into phylogroups D and B2 occur less frequently [12,18,19,20]. Porcine ExPEC are mainly distributed among phylogroups A, B1, and D and rarely among the B2 group [6]. We previously described the phylogeny of commensal *E. coli* from post-weaning piglets and sows, and our study showed significant differences in distribution of phylogroups A and B1. Among strains from piglets, phylogenetic group B1 prevailed significantly, while in sows, *E. coli* in group A were the most frequent. The strains belonging to phylogroup B2 are the least frequent in both groups [21]. In the literature, there is information concerning the phylogeny of porcine commensal *E. coli* based on the previous Clermont phylogenetic typing method [22], but there is a lack of analyses using the new, revised Clermont scheme [15]. Changes in the designation of phylogenetic type are important in identifying new groups of analyzed strains. Phylogenetic typing by the new scheme results in re-distribution of some of the identified isolates into new groups from their original designation and reveals a more complex phylogenetic structure of the analyzed strains [15,23]. The revised Clermont phylotyping method is a useful tool for subtyping various ExPEC lineages, allowing differentiation of *E. coli* strains with higher or lower virulence potential [23]. Reclassification of porcine *E. coli* from our collection may show the differences between the phylogenetic pattern characteristic for post-weaning piglets and sows and distinguish the phylogroups with different pathogenic potential.

Infections caused by ExPEC strains occur worldwide and are associated with great economic cost [24]. ExPEC is an important pathogen in the swine industry, in several countries, responsible for substantial economic losses [25,26,27]. Porcine ExPEC is an important pathogen causing UTI—a significant cause of death in adult animals [6]. Other diseases induced by these strains are coliform mastitis, meningitis, pneumonia, arthritis, and septicemia [6,28]. Similar virulence profiles and serogroups have been detected both in porcine and human ExPEC, suggesting that there is a cross-infection possibility between human and pigs, and that these pathogens may have strong zoonotic potential [6,28,29]. Therefore, porcine ExPEC poses a high risk for food safety and public health security [30,31,32]. As mentioned above, the line between commensals and ExPEC is very thin. An example is *E. coli* clonal complex 10 (CC10), which is a commensal gastrointestinal inhabitant in pigs, other food-production animals, wild animals, and humans. On the other hand, certain strains of *E. coli* CC10 are known to cause extra-intestinal disease in pigs, dogs, and humans [6,33,34]. Therefore, it is important to investigate the presence of VGs, including in the commensal flora of food animals. It may give us information on the extent of the reservoir of VGs carried by commensal *E. coli* from pigs.

The scope of this study was to investigate the extended phylogeny and extraintestinal virulence potential of commensal *E. coli* isolated from two groups of healthy pigs (post-weaning piglets and sows). We aimed to compare the phylogenetic assignment using the revised (this study) and previous (the earlier data [21]) Clermont phylogenetic typing methods. The purpose was also the identification of the VGs typical for extraintestinal pathogens, their distribution in the phylogenetic structure, and genotypic analysis of two fimbrial operons for type I and P fimbriae among *E. coli* from piglets and sows.

## 2. Materials and Methods

### 2.1. Sample Collection, Isolation, and Identification of Escherichia Coli (E. coli)

The samples were derived from a pig breeding farm in Lubuskie Province (Western Poland) and were collected in 2011. Two groups of healthy pigs were included in the study. The first one consisted of 49 post-weaning piglets (6 weeks and 8 weeks old), and the second comprised 50 sows (5 months and 7 months old). Fresh fecal samples were collected only once, and *E. coli* was selected as described previously [21]. Briefly, fecal samples were plated on membrane Fecal Coliform (mFC) chromogenic agar (Merck, Darmstadt, Germany) and incubated at 44 °C for 24 h. The blue colonies typical for fecal coliforms were subcultured on the MacConkey’s agar (Merck) and incubated at 37 °C for 24 h. The typical lactose fermenting colonies were randomly selected and identified by biochemical tests: Indole production, Methyl red reaction, Voges-Proskauer test, and Citrate utilization (IMVC). This study involved the same set of isolates as in the earlier study [21], namely 274 unique *E. coli*, from one to four per animal, 110 isolated from post-weaning piglets, and 164 from sows. The DNA extraction was carried out using the thermal cell lysis method, 1.5–3 µL of the boiled bacterial supernatant was used as a template in all the PCR reactions.

### 2.2. Extended Phylogenetic Grouping

Phylogenetic analysis was performed using the revised Clermont phylogenetic typing method described by Clermont et al. in 2013. *E. coli* isolates were assigned to phylogroups A, B1, B2, D, and F using new quadruplex PCR. Additional specific screening, in a separate PCR reaction, was carried out to classify *E. coli* into phylogroups C and E and clade I [15,35,36,37]. Escherichia coli Reference (ECOR) collection strains (Institut Pasteur Collection, Paris, France) were examined in all PCR reactions as positive and negative controls, respectively.

### 2.3. Virulence Genes (VGs) and Fimbrial Operons Genotyping

The *E. coli* isolates were examined for the presence of the following extraintestinal VGs, representing five functional categories: (1) adhesins: *fimH* (type 1 fimbriae), *papAH*, (P fimbriae), and *sfaS* (S fimbriae); (2) iron acquisition: *fyuA* (yersiniabactin siderophore receptor), *iutA* (aerobactin siderophore receptor), *iroN* (salmochelin siderophore receptor), *ireA* (iron-regulated element, siderophore receptor); (3) protectins: *kpsMTII*, *kpsMT* K1, *kpsMT* K2, *kpsMT* K5 (group II capsule with K1, K2 and K5 variants), *kpsMTIII* (group III capsule), *ompT* (outer membrane protein, protease), *traT* (serum resistance-associated outer membrane protein), and *iss* (increased serum survival); (4) toxins: *cnf1* (cytotoxic necrotizing factor 1) and *hlyA* (alpha hemolysin); (5) biofilm formation: *agn43*, *agn43* a, and *agn43* b (antigen 43, common, and alleles a and b). The VGs in functional categories were chosen according to the previous published data as typical for ExPEC strains [38,39,40,41,42,43,44,45,46]. The isolates positive for the adhesin genes *fimH* and *papAH* additionally were screened for the presence of the other operon genes: (1) *fimB*, *fimE*, *fimA*, *fimI*, and *fimC* and (2) *papC*, *papEF*, *papG*, and its variants (*G*I, *G*II and *G*III), essential for expression of type 1 and P fimbriae, respectively. Multiplex or simplex PCR-based genotyping was performed with primers and conditions previously described [38,39,40,41,42,43,44,45,46]. The PCR amplification mixture in a volume of 25 μL contained: buffer solution (Thermo Scientific, Waltham, MA, USA), 2.5 mM MgCl_2_ (Promega, Madison, WI, USA), 0.5 mM of each dNTP (Promega), 0.2 μM of each primer (IDT, Coralville, IA, USA), 1 U of Dream Taq Green DNA Polymerase (Thermo Scientific), and 3 μL of DNA template. *E. coli* strains from the ECOR collection (Institut Pasteur Collection, Paris, France), Polish Collection of Microorganisms (Institute of Immunology and Experimental Therapy, Polish Academy of Sciences, Wroclaw, Poland), and from our collection of human fecal strains, known to possess (validated by sequencing) or lack the genes of interest, were examined in all PCR reactions as positive and negative controls, respectively. The PCR products were separated in 1.5% or 2% agarose gel electrophoresis and stained with ethidium bromide.

### 2.4. Statistical Analysis

The presence of VGs was categorized as 1 = yes and 0 = no. The relations between the presence of VGs and two groups of pigs were described using the logistic regression mixed model. In this approach, each individual with at least one strain positive for a virulence gene was considered as an individual with *E. coli* positive for these factors. The mixed logit model approach takes into account the correlated nature of the isolates derived from one pig.

In each case, we assume the mixed logistic regression with one random effect per animal (pig). Let *y_ij_* = 1 if the *j*th isolate derived from the *i*th pig has the virulence gene and *y_ij_* = 0 otherwise. In this case *i* = 1, …, 99 and *j* = 1, …, *n_i_* where *n_i_* is the number of isolates derived from the *i*th pig. We consider the following model:

*logit*(*π_ij_*) = *β*_0_ + *β*_1_*x_ij_* + *b_i_* (1)

where *π_ij_* = *P* (*y_ij_* = 1 | *b_i_*) is the conditional probability of gene presence for an individual random effect *b_i_* associated with the *i*th pig, assumed to have a normal distribution with mean zero and variance *σ*^2^*_b_*. The fixed effect, *x_ij_*, is the categorical variable that can take two levels: piglets or sows, which is the reference category, and *β*_0_, *β*_1_ are regression coefficients.

The evaluations of the frequency of the gene combinations within type 1 and P fimbriae operons among the *E. coli* isolates from piglets and sows were tested using the chi-squared test for proportions or Fisher’s exact test for proportions. Fisher’s exact test was used when the assumptions of the chi-square test did not hold. The null hypothesis assumes that the proportions in isolates from piglets and sows are equal. The alternative hypothesis is one-sided and assumes that the proportion in one group of animals (piglets or sows) was lower or higher than in the other, as appropriate. In order to control the number of false positive results in a series of tests for comparing two proportions the method of false discovery rate (FDR) was used. The FDR Benjamini-Hochberg procedure (1995) allowed us to adjust the p-value in multiple testing. An analogous approach was used to compare the frequency of the *E. coli* isolates with VGs within phylogenetic groups.

In order to measure the strength of the associations for the cross tabulation of VGs of the *E. coli* isolates from both piglets and sows, the Goodman and Kruskal tau coefficient was calculated.

For all the statistical tests, the level of statistical significance was defined as 0.05. The statistical analyses were performed using the program R (R Core Team) [47,48,49].

## 3. Results

### 3.1. Extended Phylogenetic Structure of E. coli

**Figure 1:**
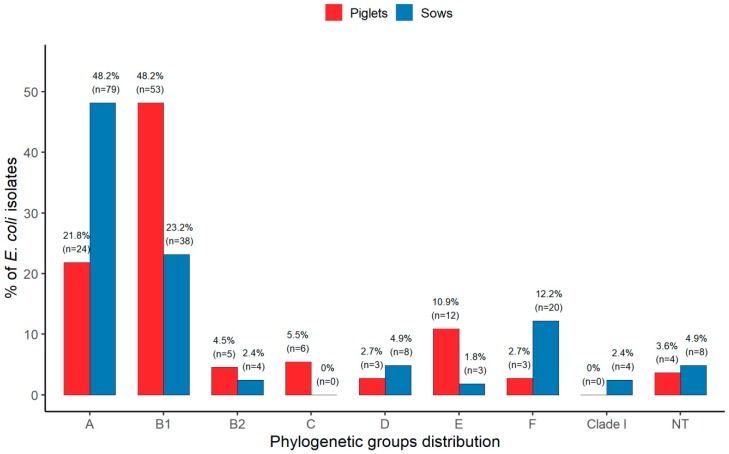
Extended phylogenetic structure of the *Escherichia coli* (*E. coli*) isolates derived from piglets and sows. Note: NT— not typeable.

Extended phylogenetic group classification revealed that *E. coli* isolates derived from piglets and sows differed significantly in their phylogenetic structure (*p* = 0.0005) (Figure 1). The majority of *E. coli* from piglets (48.2%) belonged to phylogroup B1, followed by A (21.8%), the phylogroups B2, C, D, E, and F were identified at a lower frequency. None of these isolates were assigned to clade I, and 3.6% of isolates did not belong to any known phylogroup and therefore were assigned as NT (not typeable). Among the *E. coli* isolates from sows, phylogroup A was the most frequent (48.2%), followed by B1 (23.2%), while phylogroups B2, D, E, F, and clade I occurred with a lower frequency. None of the isolates belong to group C and 4.9% were classified as NT. Phylogenetic groups B1, C, and E were significantly more frequent in the isolates from piglets as compared to sows, *p* < 0.0001, *p* = 0.009, and *p* = 0.005, respectively. Conversely, phylogroups A and F significantly prevailed among the isolates from sows compared to piglets, *p* < 0.0001 and *p* = 0.01, respectively.

All isolates were analyzed individually using the previous Clermont phylogenetic typing method (in earlier study [21]) and revised Clermont protocol (this study). The comparison of the previous and revised classification of the *E. coli* isolates is presented in Supplementary Table S1. The new phylogenetic classification for most isolates is consistent with the original one. Designation change rates were similar for both groups of isolates and were 27.2% and 31% among *E. coli* from piglets and sows, respectively. For the *E. coli* from piglets, the dominant phylogroup by the old typing scheme was B1 and after the revision, this category still dominated, while the greatest changes were noted for A to C re-classification (5.5%) and D to E (10.9%). The majority of isolates from sows were typed as phylogroup A according to the old scheme and this category also dominated after revision, whereas a considerable number of isolates were re-classified from A to B1 (5.5%) and from D to F (11.6%).

### 3.2. Prevalence of VGs

To assess the virulence potential of commensal *E. coli* isolates from piglets and sows, we screened for a total of 15 genes that have been associated with extraintestinal disease caused by ExPEC. Each of these genes was present in at least one isolate. *E. coli* from piglets carried between 1 and 12 VGs, with a mean of 4.6 VGs per isolate. The isolates derived from sows possessed between 0 and 10 VGs, with a mean of 2.9 VGs per isolate. According to the definition (Johnson et al. [10]) 19 (17.3%) of E. coli isolates from piglets and 13 (7.9%) from sows were classified as ExPEC. One gene in the adhesins category (*sfaS*) and two genes from the toxins category (*cnf1* and *hlyA*) were not detected in any of the isolates from sows. The most frequent gene from the adhesins category was *fimH*, with rates of 97.3% and 86.6% for isolates from piglets and sows, respectively. The iron acquisition category was most often represented by the *iutA* gene, which occurred with the frequency of 50.9% and 20.1% for *E. coli* from piglets and sows, respectively. Among the protectins category, the *traT* gene was the most prevalent with rates of 85.5% and 54.9% for isolates from piglets and sows, respectively. For the *kpsMTII* gene, the K2 variant was the most frequent (7.3%) among the isolates from piglets but K5 (6.1%) was most frequent in *E. coli* from sows. The *cnf1* gene occurred more often (4.5%) than the *hlyA* gene (1.8%) in the toxins category, among *E. coli* from piglets. In the biofilm formation category, alleles a and b of the *agn43* gene were not frequent. The allele *agn43*a showed higher rates than allele *agn43*b with 4.5% and 1.8% for isolates from piglets and sows, respectively. The majority of the tested genes, 14/15, occurred more frequently in isolates from piglets than from sows, with statistically significant differences for seven VGs: *fimH*, *papAH*, *iutA*, *iroN*, *ompT*, *traT*, and *iss*. The exceptions were the *kpsMTIII* gene and K1 variant of the *kpsMTII* gene, which were detected less frequently among isolates from piglets. Table 1 shows these results in detail.

### 3.3. Distribution of VGs according to Phylogenetic Groups

Analysis of the distribution of VGs within phylogenetic groups revealed that there were more statistically significant differences among isolates from piglets than among isolates from sows (Table 2). The mean number of VGs for each phylogroup in isolates from piglets was: A—4.6, B1—4, B2—10.6, C—8, D—4.7, E—2.6, F—7.7, and NT—2.5. The mean number of VGs for each phylogroup among isolates from sows was: A—2.5, B1—3.6, B2—3.8, D—2.1, E—2.7, F—2.9, clade I—1.8, and NT—5. Generally, in the *E. coli* from piglets, the frequencies of the particular genes in the iron acquisition, protectins, and biofilm formation categories were significantly lower (*p* < 0.05) in the isolates of groups B1 and E, whereas the isolates of groups B2 and C harbored the virulence determinants in adhesins, iron acquisition, protectins, and toxins categories significantly more frequently (*p* < 0.05). Moreover, isolates of group F carried VGs in the iron acquisition and protectins categories more frequently, but due to the small number of isolates, the differences were not statistically significant. Among the *E. coli* from sows, the isolates of group A harbored the VGs in adhesins, iron acquisition, and protectins categories significantly less frequently (*p* < 0.05). Two genes from the adhesins and iron acquisition categories occurred significantly more frequently (*p* < 0.05) in the isolates of group B1. Only one gene from the iron acquisition category was significantly associated (*p* < 0.05) with phylogroup B2.

### 3.4. Association between VGs

**Figure 2:**
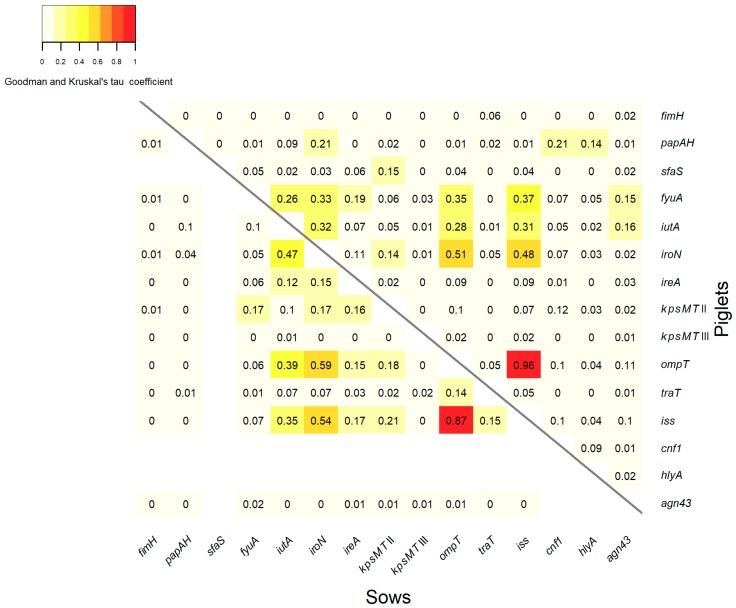
Statistical association between VGs of the *E. coli* isolates derived from piglets (the part above the diagonal) and sows (the part under the diagonal). No values were introduced in the case of undetected genes.

The statistical analysis of the association between the VGs of the *E. coli* isolates is shown in Figure 2. Similar associations occurred in both groups of isolates from piglets (Figure 2, the part above the diagonal) and from sows (Figure 2, the part under the diagonal). Very strong associations were found between the genes *ompT* and *iss*, with association coefficients of 0.96 and 0.87 in the isolates from piglets and sows, respectively. Strong associations were observed between the genes *iroN* and *ompT*, with the coefficients of 0.51 and 0.59 for *E. coli* from piglets and sows, respectively. The genes *iroN* and *iss* were also positively associated, with the coefficients of 0.48 and 0.54 among the *E. coli* from piglets and sows, respectively. Moderate associations were identified between the genes *iutA* and *iroN*, with the association coefficients of 0.32 and 0.47, and also between *iutA* and *iss* with coefficients of 0.31 and 0.35 for the isolates from piglets and sows, respectively. Additionally, in the group of isolates from piglets, moderate associations were found between the gene *fyuA* and the genes *iroN*, *ompT* and *iss*, with coefficients of 0.33, 0.35, and 0.37, respectively. Among *E. coli* from sows, a moderate association between the genes *iutA* and *ompT* with a coefficient of 0.39 was observed. Weak associations, with coefficients ≤ 0.3, occurred between the remaining VGs in both groups of isolates from piglets and sows. Generally, significant associations were observed between the genes within the iron acquisition and protectins categories or between these two categories.

### 3.5. Genotypic Analysis of Fimbrial Operons for Type 1 and P Fimbriae

**Figure 3:**
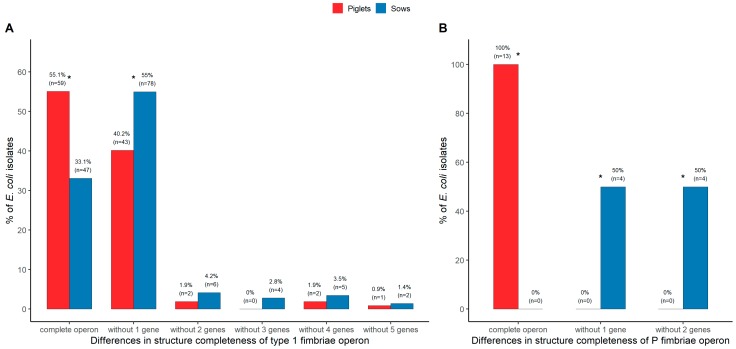
Frequency of the gene combinations within the structure of (**A**) type 1 and (**B**) P fimbriae operons among the *E. coli* isolates from piglets and sows. Note: The gene combinations within the structure of type 1 fimbriae operon: Complete operon: *fimB-fimE-fimA-fimI-fimC-fimH*; Without 1 gene: *fimB-fimE-fimI-fimC-fimH*; Without 2 genes: *fimB-fimI-fimC-fimH*, *fimB-fimE-fimC-fimH*, *fimE-fimI-fimC-fimH*; Without 3 genes: *fimB-fimC-fimH*, *fimB-fimA-fimH*, *fimI-fimC-fimH*, *fimB-fimE-fimH*; Without 4 genes: *fimB-fimH*, *fimC-fimH*, *fimI-fimH*; Without 5 genes: *fimH*. The gene combinations within the structure of P fimbriae operon: Complete operon: *papAH-papC-papEF-papG*; Without 1 gene: *papAH-papC-papEF*; Without 2 genes: *papAH-papC*. * Statistically significant.

The genes *fimH* and *papAH* are considered to be genetic markers for type 1 and P fimbriae, respectively. The further analysis of this study encompassed the detection of the other crucial genes of type 1 and P fimbriae operons among the isolates positive for the *fimH* and *papAH* genes, respectively. The complete set of six tested genes of the type 1 fimbrial operon occurred significantly more frequently in the isolates from piglets (55.1%), as compared to sows (33.1%), *p* < 0.0001. Altogether, 13 various gene combinations of this operon were detected, 6 and 12 among the isolates from piglets and sows, respectively. The combination without one gene (*fimA*) was the most frequent in both groups of isolates, but significantly prevailed in *E. coli* from sows (54.9%), as compared to piglets (40.2%), *p* = 0.0148. The other gene combinations without two, three, four, and five genes within the type 1 fimbrial operon occurred less frequently (Figure 3A). Regarding the P fimbrial operon, the complete set of four tested genes was present in all *E. coli* positive for the *papAH* gene derived from piglets, but were not detected in any of the isolates from sows, *p* < 0.0001. The *PapG* gene encodes the adhesin at the tip of the P fimbriae and may be represented by one of three alleles: *papGI*, *papGII*, or *papGIII*. This gene occurred only among *E. coli* from piglets, where the allele *papGIII* was identified most often (84.6%). The other two alleles, *papGI* and *papGII*, were detected less frequently (7.7% for both). Among the isolates from sows, two P fimbrial operon gene combinations were found, without one (*papG*) or two (*papEF* and *papG*) tested genes (Figure 3B).

## 4. Discussion

The present study examined for the first time, to our knowledge, the extended phylogenetic structure of commensal *E. coli* derived from two age groups of pigs (weaned piglets and sows) using the revised Clermont phylogenetic typing method [15]. Moreover, our study focused on the analysis of the extraintestinal virulence potential of these isolates. The results allowed us to gain a better understanding of the genetics of this population and the association between virulence gene carriage and phylogenetic type.

Our previous study on the same set of *E. coli* isolates used the old Clermont typing scheme and showed the opposite distribution of phylogroups A and B1 among *E. coli* from piglets and sows. Phylogroup A dominated in isolates from sows, followed by B1 and D, while among *E. coli* from piglets, phylogroup B1 was the most frequent, followed by A and D. The least frequent in isolates from piglets and sows was phylogroup B2 [21]. The current study reclassified the basic phylogenetic structure using the revised Clermont protocol. The analysis revealed more complex phylogeny in both groups of isolates from piglets and sows. The main phylogenetic pattern of the new classification remains in agreement with the old one, with significant differences in distribution of phylogroups A and B1, where B1 group dominated among *E. coli* from piglets and A in isolates from sows. Moreover, phylogenetic groups C and E were significantly more frequent in the isolates from piglets. Conversely, phylogroup F significantly prevailed among the isolates from sows. One of the most important factors influencing the differences in phylogenetic structure may be the maturation of the digestive tract during growth of piglets and the replacement of milk with solid food in the diet [16,50]. Most changes were observed for conversion from A to C, D to E, and D to F in isolates from piglets. Among *E. coli* from sows, there also appeared new phylogroups, the most frequent changes occurred from D to F, while a few isolates were reclassified into phylogroup E and clade I. There were a small number of isolates from piglets and sows, which according to the new classification, did not belong to any known phylogroup and therefore were assigned as NT. Using the new phylogenetic analysis approach, this study changed the designations of 27.2% and 31% of isolates from piglets and sows respectively, and retained about 70% of isolates in their original phylogenetic type. A significant reduction of phylogenetic group D in *E. coli* from piglets and sows can be considered the most striking reclassification result. It has been reported that the rate of reclassification is closely correlated with the species of the host and pathogenicity status of *E. coli* isolates. Among the human isolates, the changes ranged from 8.6% to 13% human fecal *E. coli* (HFEC) to 14% to 15% for neonatal meningitis *E. coli* (NMEC) and uropathogenic *E. coli* (UPEC) compared to the animal sources, where reclassification rates ranged from 21.6% for avian fecal *E. coli* (AFEC) to 53.8%, with the greatest rate of reclassification observed for avian pathogenic *E. coli* (APEC) [15,23].

Overall, the main pattern of phylogenetic designation of commensal *E. coli* from pigs in our earlier [21] and present studies is consistent with the other reports [12,18,19,20,51], namely the phylogroups A and B1 are more frequent than D and B2. There may occur a shift between the frequency of phylogroups A and B1, but these two groups together represent 70% or more of all *E. coli* isolates. The least frequent is phylogroup B2. Interestingly, the phylogenetic structure of porcine ExPEC is similar to the phylogeny of the commensal isolates. ExPEC from diseased pigs mostly belonged to phylogroups A and B1, while phylogroup B2 is usually the least numerous [6,26,52,53]. It has been reported that ExPEC isolated from piglets with septicemia, often pathogenic in experimental infections, fall into groups A and B1 [54]. These data are an obvious contrast with the fact that most ExPECs isolated from humans or companion animals belong to groups B2 and D [55].

Our study indicated that commensal *E. coli* from pigs carried the broad range of VGs typical for ExPEC, representing five functional categories: adhesins, iron acquisitions, protectins, toxin, and biofilm formation. According to the definition (Johnson et al. [10]) 17.3% of E. coli isolates from piglets and 7.9% from sows were classified as ExPEC, despite the fact that they were derived from healthy animals. The other isolates in our collection also harbored VGs associated with the ability to cause extraintestinal disease, not only in swine but also in humans [4,26]. The results showed significant differences between isolates from piglets and sows. The majority of VGs were identified more frequently in isolates from piglets than from sows—seven of them: *fimH*, *papAH*, *iutA*, *iroN*, *ompT*, *traT*, and *iss*—with statistically significant differences. Such results indicated the greater proportion of potential ExPEC among commensal *E. coli* from weaned piglets than from sows, which is consistent with earlier studies [21,56,57]. Weaning is the crucial moment in the piglet’s life. It is connected with stress during separation from the dam and with the changes in the diet, which causes environmental instability [50]. In consequence, a higher number of commensal *E. coli* acquired with the feed can settle themselves in the intestine. When the microbiota is stabilized, in older pigs, only the best adapted *E. coli* remains in the intestine [57].

The other aspect of possessing VGs is associated with gut colonization. Some specific combinations of genes could promote adaptations to a given environment. Extraintestinal VGs encoding adhesins, iron capture systems, toxins, and protectins have been correlated with successful gut colonization in humans, dogs, and piglets [1,58,59,60]. A study concerning colonization of *E. coli* in a sow’s and her piglets’ intestines showed that clones which were dominant at least once throughout the sampling period tended to have more VGs. *E. coli* bacteria with higher numbers of VGs, particularly of iron acquisition genes, were detected in more piglets and more often in the piglets, even after weaning. VGs typical for ExPEC promote successful colonization of the intestine in pigs [60].

Many studies have indicated that the most highly virulent human ExPEC strains, which cause urinary tract infection, neonatal septicemia, or meningitis, belong to group B2 or D and that the strains of these phylogroups harbor more virulence factors than the strains of the A and B1 groups [7,13]. Moreover, it has been reported that porcine ExPEC B2 isolates contained more virulence-associated genes than non-B2 isolates and these isolates had the strongest pathogenicity in a mouse infection model [26]. Another study found no significant differences in means (ranges) of VGs across different phylogenetic groups among ExPEC from pigs [6]. An earlier study also revealed that some strains of phylogenetic groups A, B1, and D were able to kill the mice and possessed virulence determinants [54]. Our results showed that isolates of phylogroups B2, C, and to a lesser extent F, (statistically not significant for F) from piglets carried VGs significantly more often, whereas isolates in phylogroups A and B1 did so less frequently. The analysis of the distribution of VGs within phylogenetic groups among *E. coli* from sows revealed no significant differences, the one exception being isolates of phylogroup A, which carried VGs significantly less frequently. Our results demonstrating the virulence potential of group B2 in isolates from piglets are consistent with those presented in the first, above-mentioned report concerning ExPEC [26]. On the other hand, it was also reported that VGs were distributed among four main phylogroups without significant differences in commensal porcine isolates [12], which is consistent with our analysis concerning *E. coli* from sows. The advantage of our study is the analysis of extended phylogenetic structure, which allows for more accurate designation of isolates to phylotypes. The appearance of phylogroup C in isolates from piglets was a consequence of redistribution of *E. coli* from group A, and, what is interesting, the group C isolates harbored VGs more frequently than the isolates in other groups. The redistribution of isolates from phylogroup D in piglets to group F also distinguished *E. coli* with higher virulence potential, whereas the isolates redistributed from group D to E carried VGs less frequently. Our results are partially consistent with the study concerning extended phylotyping of human and avian ExPEC and commensal *E. coli* [23]. The aforementioned study reported that redistribution of APEC isolates from A to C is connected with distinction of isolates with the lower number (group A) and higher number (group C) of pathogenicity-, resistance-, replicon-, and pathogenicity island-associated genes. The isolates that became reclassified as F from their original D designation appeared to harbor higher levels of these genes and indicated that APEC designated as the F phylogenetic type are probably highly pathogenic.

Mobile genetic elements transmitted via horizontal gene transfer play an important role in the evolution of *E. coli*. Most ExPEC VGs are clustered together on mobile genetic elements, usually on pathogenicity islands (PAI) or virulence plasmids, in unique organization. The analysis of associations between VGs may indicate which genes tend to occur in the same genetic element. Our analysis revealed that in commensal *E. coli* from piglets and sows there occurred similar associations between VGs, which suggests that the same genetic elements are present in *E. coli* isolates from piglets and sows. The most significant associations were identified between the genes within iron acquisition and protectins categories or between these two categories. Particularly the genes *ompT* and *iss*, *iroN* and *ompT*, and also *iroN* and *iss*, are strongly associated. These genes usually occur together in the conserved virulence plasmidic (CVP) region, typical for the ExPEC virulence-associated plasmids [61].

Type 1 and P fimbriae are the major virulence factors of the UPEC strains. Type 1 fimbriae mediate adhesion, invasion, and intracellular formation of biofilm-like structures and are responsible primarily for bladder infection. P fimbriae are mainly related to pyelonephritis [62]. The *fimH* gene is the most widespread virulence gene among the *E. coli* from piglets and sows, but our analysis of six genes forming the fimbrial operon demonstrated that among isolates positive for the gene *fimH*, there occurred a large variety of incomplete combinations of these genes. The complete set of six tested genes of the type 1 fimbrial operon occurred significantly more frequently in the isolates from piglets than from sows. The combination without one gene (*fimA*) was the most frequent in both groups of isolates, but significantly prevailed in *E. coli* from sows. Incompleteness of this operon is rather common in commensal *E. coli* from pigs, reaching 44.9% and 66.9% among isolates from piglets and sows, respectively. The previous studies indicated that many human commensal isolates positive for the *fimH* gene showed a lack of type 1 fimbriae due to the deletion or lack of the expression of one or more of the type 1 fimbriae operon genes [63]. Such results suggest that the *fimH* gene is not a good marker of type 1 fimbriae, much better would be the *fimA* gene. The analysis of the completeness of the P fimbrial operon revealed that all *E. coli* positive for the *papAH* gene were derived from piglets that possessed the complete set of four tested genes. The *papG* gene was represented the most often by the allele *papGIII* mainly associated with lower urinary tract infections (cystitis) [64]. The complete P fimbrial operon was not detected in any of the isolates from sows, indicating that these *E. coli* cannot express P fimbriae.

It has been reported that porcine commensal *E. coli* isolates carried large numbers of virulence-associated genes. Some of these isolates belong to ST131 and ST117, representative of pandemic ExPEC clones that cause hospital- and community-acquired infections in humans worldwide [12]. Moreover, the clonal complex 10 (CC10) was identified as the predominant clonal group within the collection of porcine commensal *E. coli* from healthy Australian food-production pigs. CC10 is a resident of the intestinal tract of humans, food-production animals, companion animals, and wild animals. *E. coli* CC10 members frequently carried VGs and they are increasingly reported as human and animal extraintestinal pathogens [6,12,33,34]. Our results and above-mentioned reports [12,34] suggest that commensal *E. coli* from pigs may have zoonotic potential associated with a specific clonal lineage within phylogenetic groups.

## 5. Conclusions

This study analyzed the extended phylogenetic structure and extraintestinal potential of commensal *E. coli* isolates from post-weaning piglets and sows. Application of the revised Clermont phylogenetic typing method revealed more complex phylogeny and significant differences in the proportion of phylogroups in both pools of isolates from piglets and sows. Particularly the revised phylogenetic assignment of A and D phylogroup isolates allowed us to distinguish *E. coli* with significantly higher (transition from A to C and from D to F) or lower (transition from D to E) virulence potential in isolates from piglets. The analysis of the VG frequencies showed that commensal *E. coli* from pigs, particularly from post-weaning piglets, constitute a substantial reservoir of VGs typical for ExPEC. The VGs preferentially accumulated in the isolates of phylogroups B2, C, and to a lesser extent F, from piglets, while in isolates from sows, VGs were distributed more evenly. On the other hand, generally in the phylogenetic structure of commensal *E. coli* from pigs, group B1 prevailed in piglets and group A in sows. Knowing that ExPEC from diseased pigs mostly belong to phylogroups A and B1, we cannot exclude that commensal strains of groups A and B1 may also pose a potential threat of extraintestinal infection. The probability of expression of type 1 and P fimbriae resulting from the presence of the complete operons was significantly higher in isolates from piglets. Recently, ExPEC infections have become more common in the pig industry [25,26,27], so it is important to understand the population structure of porcine commensal *E. coli*. We should also be aware of the reservoir of VGs carried by commensal *E. coli* from pigs in terms of the public health threat. This study provides a good starting point for further, more detailed genomic investigation.

## Figures and Tables

**Table 1:** Frequency of virulence genes (VGs) by functional categories among *E. coli* isolates from piglets and sows. The test of statistical significance for fixed effect from the mixed logistic models with VG as the response and the animal as the independent variable.

Functional Category VG	Number (%) of *E. coli* Isolates with VGs		*p*-Value
	Piglets	Sows	
	N = 110	N = 164	
Adhesins			
*fimH*	107 (97.3)	142 (86.6)	0.0065 *
*papAH*	13 (11.8)	8 (4.9)	0.0449 *
*sfaS*	2 (1.8)	0	-
Iron acquisition			
*fyuA*	29 (26.4)	23 (14.0)	0.0931
*iutA*	56 (50.9)	33 (20.1)	<0.0001 *
*iroN*	43 (39.1)	29 (17.7)	0.0012 *
*ireA*	7 (6.4)	5 (3.0)	0.2313
Protectins			
*kpsMTII*:	12 (10.9)	10 (6.1)	0.2867
-K1	4 (3.6)	9 (5.5)	0.5026
-K2	8 (7.3)	9 (5.5)	0.7091
-K5	7 (6.4)	10 (6.1)	0.9264
*kpsMTIII*	1 (0.9)	2 (1.2)	0.9971
*ompT*	36 (32.7)	28 (17.1)	0.0175 *
*traT*	94 (85.5)	90 (54.9)	<0.0001 *
*iss*	35 (31.8)	25 (15.2)	0.0143 *
Toxins			
*cnf1*	5 (4.5)	0	-
*hlyA*	2 (1.8)	0	-
Biofilm formation			
*agn43*:	59 (53.6)	87 (53.0)	0.9275
-a	5 (4.5)	3 (1.8)	0.4890
-b	2 (1.8)	2 (1.2)	0.6874

* Statistically significant.

**Table 2:** Distribution of VGs in extended phylogenetic structure. The frequency of virulence genes among a particular *E. coli* phylogroup was compared to its prevalence in a group consisting of the isolates of all the other phylogenetic groups using the appropriate test for proportions.

VG	Number (%) of *E. coli* Isolates with VGs within Phylogenetic Groups															
	Piglets								Sows							
	A	B1	B2	C	D	E	F	NT	A	B1	B2	D	E	F	Clade I	NT
	n = 24	n = 53	n = 5	n = 6	n = 3	n = 12	n = 3	n = 4	n = 79	n = 38	n = 4	n = 8	n = 3	n = 20	n = 4	n = 8
**Adhesins**																
fimH	23 (95.8)	51 (96.2)	5 (100)	6 (100)	3 (100)	12 (100)	3 (100)	4 (100)	77 (97.5)	38 (100)	2 (50.0)	5 (62.5)	3 (100)	5 (25.0)	4 (100)	8 (100)
papA	0	9 (17.0)	4 (80.0)	0	0	0	0	0	0	4 (10.5)	1 (25.0)	1 (12.5)	0	2 (10.0)	0	0
sfaS	1 (4.2)	0	1 (20.0)	0	0	0	0	0	0	0	0	0	0	0	0	0
**Iron acquisition**																
fyuA	9 (37.5)	8 (15.1)	5 (100)	6 (100)	0	0	1 (33.3)	0	11 (13.9)	6 (15.8)	2 (50.0)	0	1 (33.3)	0	0	3 (37.5)
iutA	13 (54.2)	23 (43.4)	5 (100)	6 (100)	2 (66.7)	4 (33.3)	3 (100)	0	8 (10.1)	9 (23.7)	4 (100)	2 (25.0)	0	7 (35.0)	0	3 (37.5)
iroN	9 (37.5)	18 (34.0)	5 (100)	6 (100)	1 (33.3)	0	3 (100)	1 (25.0)	7 (8.9)	13 (34.2)	1 (25.0)	0	0	5 (25.0)	0	3 (37.5)
ireA	3 (12.5)	2 (3.8)	1 (20.0)	1 (16.7)	0	0	0	0	2 (2.5)	1 (2.6)	0	0	0	1	0	1 (12.5)
**Protectins**																
kpsMTII	2 (8.3)	1 (1.9)	4 (80.0)	0	1 (33.3)	1 (8.3)	2 (66.7)	1 (25.0)	2 (2.5)	3 (7.9)	0	1 (12.5)	0	1 (5.0)	0	3 (37.5)
kpsMTIII	0	1 (1.9)	0	0	0	0	0	0	2 (2.5)	0	0	0	0	0	0	0
ompT	6 (25.0)	14 (26.4)	5 (100)	6 (100)	2 (66.7)	0	3 (100)	0	7 (8.9)	11 (28.9)	0	0	0	7 (35.0)	0	3 (37.5)
traT	19 (79.2)	49 (92.5)	5 (100)	5 (83.3)	2 (66.7)	9 (75.0)	3 (100)	2 (50.0)	36 (45.6)	25 (65.8)	2 (50.0)	5 (62.5)	2 (66.7)	13 (65.0)	1 (25.0)	6 (75.0)
iss	6 (25.0)	14 (26.4)	5 (100)	6 (100)	1 (33.3)	0	3 (100)	0	7 (8.9)	9 (23.7)	0	0	0	6 (30.0)	0	3 (37.5)
**Toxins**																
cnf1	0	1 (1.9)	3 (60.0)	1 (16.7)	0	0	0	0	0	0	0	0	0	0	0	0
hlyA	0	1 (1.9)	1 (20.0)	0	0	0	0	0	0	0	0	0	0	0	0	0
**Biofilm formation**																
agn43	20 (83.3)	19 (35.8)	4 (80.0)	5 (83.3)	2 (66.7)	5 (41.7)	2 (66.7)	2 (50.0)	41 (51.9)	19 (50.0)	3 (75.0)	3 (37.5)	2 (66.7)	10 (50.0)	2 (50.0)	7 (87.5)

The values significantly higher than among the other groups are indicated in yellow. The values significantly lower than among the other groups are indicated in blue.

## Supplementary Materials

The following are available online at https://www.mdpi.com/1660-4601/17/1/366/s1. Table S1: Comparison of phylogenetic structure of *E. coli* isolates from piglets and sows using previous and revised Clermont phylogenetic typing method.
