# Supplementary material for: Extended Phylogeny and Extraintestinal Virulence Potential of Commensal Escherichia coli from Piglets and Sows
Source: Int J Environ Res Public Health. 2020 Jan 6;17(1):366. doi: 10.3390/ijerph17010366 (PMC6981902; doi:10.3390/ijerph17010366)
Supplement: Supplementary file 1 [file ijerph-17-00366-s001.pdf]

Table S1. Comparison of phylogenetic structure of *E. coli* isolates from piglets and sows using previous and revised Clermont phylogenetic typing method.

	Number (%) of *E. coli* isolates within phylogenetic groups												
	Clermont Scheme 2000				Clermont Scheme 2013								
	**A**	**B1**	**B2**	**D**	**A**	**B1**	**B2**	**C**	**D**	**E**	**F**	**Clade I**	**NT**
piglets	29 (26.3)	54 (48.2)	7 (7.3)	20 (18.2)	24 (21.8)	53 (48.2)	5 (4.5)	6 (5.5)	3 (2.7)	12 (10.9)	3 (2.7)	-	4 (3.6)
sows	89 (54.3)	36 (22)	5 (3)	34 (20)	79 (48.2)	38 (23.2)	4 (2.4)	-	8 (4.9)	3 (1.8)	20 (12.2)	4 (2.4)	8 (4.9)
